# Cutaneous Disease as Sole Clinical Manifestation of Protothecosis in a Boxer Dog

**DOI:** 10.1155/2016/2878751

**Published:** 2016-02-29

**Authors:** Emmanouil I. Papadogiannakis, Emmanouil N. Velonakis, Gregory K. Spanakos, Alexander F. Koutinas

**Affiliations:** ^1^Department of Veterinary Public Health, National School of Public Health, 115 21 Athens, Greece; ^2^Small Animal Dermatology Clinic, Alimos, 174 55 Athens, Greece; ^3^Department of Applied Microbiology and Immunology, National School of Public Health, 115 21 Athens, Greece; ^4^Department of Parasitology, Entomology and Tropical Diseases, National School of Public Health, 115 21 Athens, Greece; ^5^Quality Veterinary Practice, 383 33 Volos, Greece

## Abstract

*Prototheca wickerhamii* is ubiquitous, saprophytic achlorophyllous algae that cause opportunistic infections in the dog and cat and disseminated disease usually in immunocompromised animals. In this report an uncommon case of canine cutaneous protothecosis is presented. A 6-year-old female boxer was brought in with skin lesions that consisted of nodules and generalized footpad hyperkeratosis, depigmentation, and erosion. Cytology and histopathology showed pyogranulomatous inflammation along with organisms containing round sporangia with spherical sporangiospores. PCR and sequencing identified the causal organism as* Prototheca wickerhamii*. Therapy applied in this patient with either fluconazole alone or combination of amphotericin B and itraconazole proved effective only for footpad lesions but not for skin nodules. Systemic therapy seems to be ineffective for skin nodules, at least in chronic cases of canine cutaneous protothecosis. Although canine protothecosis usually presents with the disseminated form, cutaneous disease as sole clinical manifestation of the infection may also be witnessed.

## 1. Introduction

*Prototheca* spp. are ubiquitous, saprophytic achlorophyllous algae that cause opportunistic infections in both small animals and disseminated disease actually in the immunocompromised ones [1]. In the dog, protothecosis is usually manifested as a disseminated disease [1, 2]. Three species are currently recognized within the genus* Prototheca*:* P. stagnosa*,* P. zopfii*, and* P. wickerhamii*, with the latter two being the most commonly isolated species from dogs [2, 3].

Although ulcerative colitis [4], ocular disease that may lead to sudden blindness [5, 6], and granulomatous encephalomyelitis [7] may occur as single clinical entities or in various combinations [8, 9], the disseminated form of the disease is by far the most common in the dog [3, 10, 11].

This report describes a case of cutaneous protothecosis in a dog the breed of which tends to develop granulomatous skin diseases either infectious (e.g. leishmaniosis, leproid granulomas) or sterile [12].

## 2. Case Description 

A six-year-old female boxer dog presented with a 13-month history of progressive and mildly pruritic skin lesions. The dog was current on vaccinations and deworming and was being fed on dry commercial food of high quality. Previous treatments included amoxicillin plus clavulanic acid (20 mg/Kg/12 h), cefalexin (25 mg/Kg/12 h) alone or in combination with prednisolone (0.5 mg/Kg/24 h for 1 week and then every other day) for a period of approximately 3 to 4 weeks each, but of no avail. The owner also reported that the dog had intermittently been experiencing nonambulatory lameness on the right front leg.

**Figure 1:**
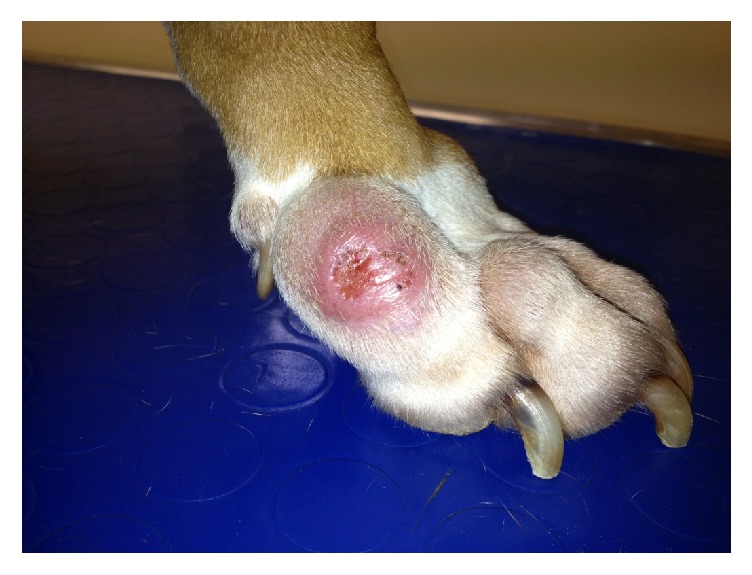
Nodules (one ulcerated) on the distal extremity and digits.

**Figure 2:**
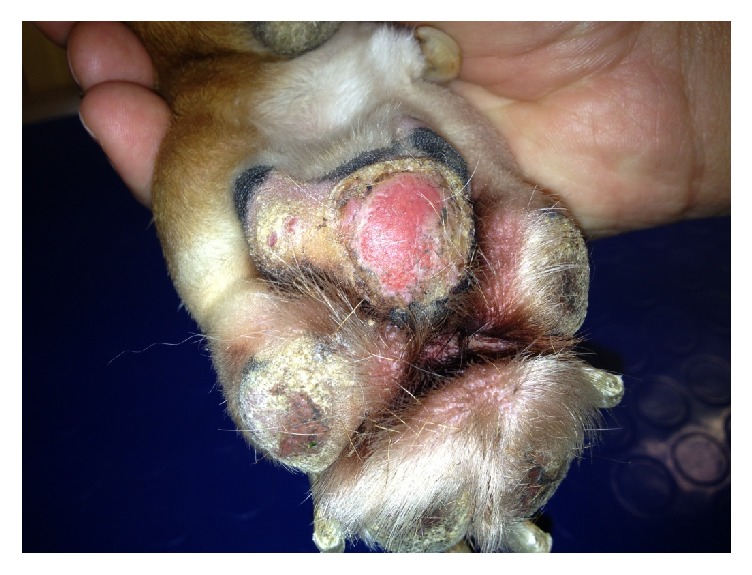
Hyperkeratosis, depigmentation, and erosions of footpads.

Physical examination of the dog upon admission revealed no abnormality. On dermatological examination, 9 ulcerated and nonulcerated skin nodules were observed, ranged from 1 to 7 cm in diameter, and distributed mainly over bony prominences of distal extremities and digits of the front legs (Figure 1), left elbow, and right hock. Other skin lesions included footpad hyperkeratosis, crusting, depigmentation, and erosions (Figure 2).

At that time the main differentials included infectious or sterile nodules and neoplasia.

Fine needle aspiration (FNA) cytology made from material obtained from nonulcerated skin nodules revealed pyogranulomatous inflammation and numerous mainly extracellular round-shaped organisms, ranging from 20 to 30 *μ*m in diameter, most containing 2 spores of approximately 10 *μ*m in diameter. A tentative diagnosis of systemic mycosis was made. Culture of FNA material in dermatophyte test medium (DTM) was performed at room temperature. Furthermore, skin biopsies were obtained from intact nodules (the owner refused footpad lesions biopsy) along with blood and urine samples for further laboratory workup and serology. Survey thoracic and abdominal radiographs were also taken but were unremarkable.

**Figure 3:**
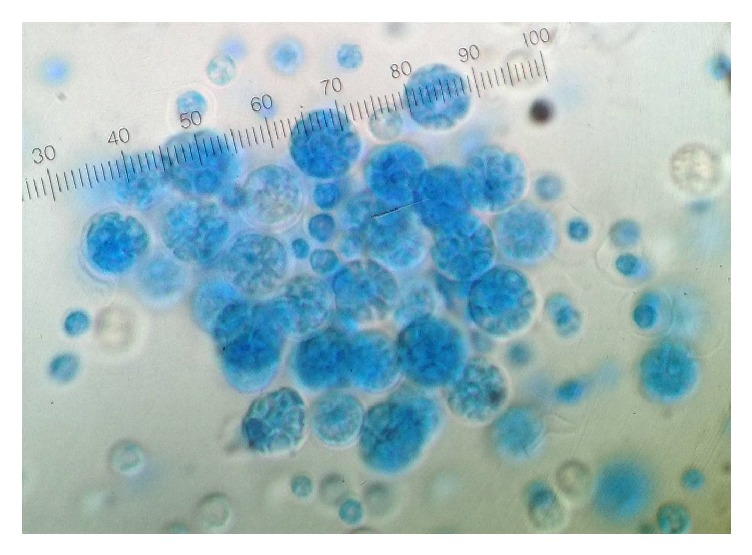
Light microscopy of lactophenol cotton blue slide preparations made of culture colonies revealed round sporangia containing spherical sporangiospores similar to those of* P. wickerhamii*.

Hematology, serum biochemistry, and urinalysis did not display any abnormalities and serology (snap ELISA, IDEXX®) for all of* Leishmania infantum*,* Ehrlichia canis*, and* Anaplasma phagocytophilum *antibodies and* Dirofilaria immitis* antigen was negative. After 4 days of incubation, smooth, creamy, yeastlike colonies were grown on DTM. Light microscopy of lactophenol cotton blue slide preparations made of these colonies revealed round sporangia containing spherical sporangiospores similar to those of* P. wickerhamii* (Figure 3).* P. zopfii* cells are oval or cylindrical in shape, producing sporangia of larger diameter (15–25 *μ*m) containing up to 20 sporangiospores. In contrast,* P. wickerhamii* cells tend to be round, forming sporangia (7–13 *μ*m) containing up to 50 spherical sporangiospores [2].

**Figure 4:**
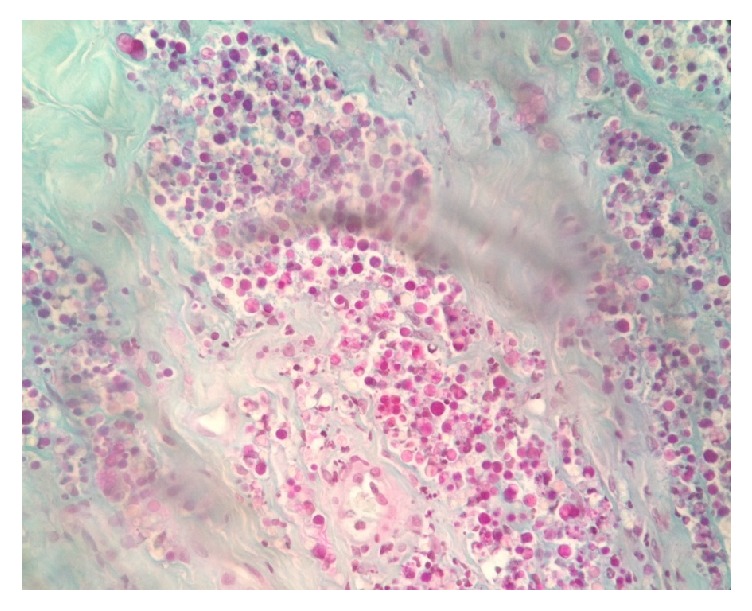
Nodular-to-diffuse, pyogranulomatous panniculitis with numerous* Prototheca*-like elements stained vividly purple with PAS stain.

Histopathology revealed nodular-to-diffuse, pyogranulomatous dermatitis and panniculitis (with lymphocytes, plasma cells, macrophages, and neutrophils) with numerous elements exhibiting* Prototheca* spp. morphology; their cell wall stained vividly purple with periodic acid Schiff (PAS) stain (Figure 4) and most of microorganisms were extracellular, either single or more often in groups, with only a few seen to be phagocytosed.

**Figure 5:**
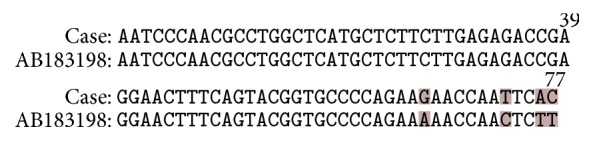
Alignment of the sequence obtained in this case to the corresponding fragment of the GenBank number AB183198 sequence. Highlighted letters indicate the differences. The image was edited with Krita 2.8.5 (https://krita.org).

Approximately 1 mm^3^ of culture material was used for DNA isolation, by employing the QIAamp Mini Kit (QIAGEN, Hilden, Germany), and following the manufacturer's instructions. A portion of the 28S rRNA gene was amplified by using already published primers [13]. The band was excised from the gel and DNA was isolated using the DNA Isolation Spin-Kit Agarose (AppliChem, Darmstadt, Germany). The isolated DNA was subsequently sequenced with the PCR primers; PCR produced a ~350 bp band. As sequencing of the complete length of PCR product was not possible, a 77 bp sequence was obtained by employing the U2 primer. Beyond that fragment the double peaks were indicative of the presence of more than 1 strain. Similar sequences were searched in the GenBank with the aid of the Web interface of Blast software which returned 9 of these sequences that belonged to* Prototheca wickerhamii* strains; the higher similarity applied to GenBank number AB183198 sequence (Figure 5). This result confirmed the diagnosis of cutaneous protothecosis due to* Prototheca wickerhamii*.

As no treatment guidelines are available, the patient was treated with oral fluconazole (10 mg/Kg twice a day), based on reported agents likely to be most useful against* Prototheca* species such as amphotericin B (AMB), fluconazole, itraconazole, and possibly terbinafine [2]. Although significant clinical improvement was witnessed in footpad lesions after one month on fluconazole, this treatment regimen did little to slow the progression of skin nodules, because* Prototheca* organisms were found on cytology. At that time fluconazole administration was withdrawn and AMB was administered twice weekly as a subcutaneous infusion using a protocol developed to treat canine cryptococcosis [14]. Specifically, 0.5 mg AMB/Kg/sc per dose was administered twice weekly in 500 mL of 0.45% NaCl/2.5% dextrose fluids. The dog was given concurrently itraconazole (5 mg/Kg/per os, once daily). Due to nephrotoxicity, AMB was withdrawn after 7 infusions and the patient is still being treated with itraconazole alone for about six months. However, although skin nodules have not been improved with this treatment regimen, footpads remain close to normal.

## 3. Discussion

Protothecosis is a very uncommon disease that has been reported in humans [15], cattle [16], cats [17], and dogs [1–11]. In Greece this is the second reported case of canine protothecosis, the other one having been associated with colonic and rectal insult [4].

A striking similarity between this case report and the largest case series of canine protothecosis ever published [2] is the overrepresentation of boxer dogs suggesting a genetic predisposition to develop the infection [18]. The increased risk to this breed of developing infectious diseases in which cellular immunosuppression plays a crucial role [2] has been witnessed in cryptococcosis [19] and leishmaniosis [12]. Due to the fact that cutaneous nodules, either ulcerated or not, and generalized footpad hyperkeratosis accompanied by depigmentation and erosion were the main constituents of the cutaneous disease in this dog and its many clinical similarities to canine leishmaniosis (*Leishmania infantum/chagasi*) [12, 20], made its exclusion with the aid of serology and cytology a diagnostic priority. Leproid granuloma (*Mycobacterium avium* complex), a top differential due to the many clinical similarities and the breed of the dog [21], was also ruled out with both cytology and histopathology.

The fact that the affected boxer was female complies with what has already been reported on several occasions [2]. Gender predisposition remains an unclarified issue and its association with female hormonal level has been argued [2].

Cutaneous protothecosis in dogs without other organ involvement is uncommon [22]. Colonic and rectal injury is the most consistent feature of* Prototheca* infection, even in the absence of overt colitis [2, 18]. Subclinical colitis would not be ruled out without colonoscopy and histopathology which were denied by the owner; his main concern was the skin lesions of his dog.

Footpad erosion and ulceration have been reported to occur in canine protothecosis [22], but not hyperkeratosis and depigmentation, although footpad biopsies should have been obtained in this case to confirm the presence of* Prototheca* organisms in those lesions. Portal of entry of* Prototheca* organism is thought to be skin wounds or colonic mucosa in disseminated disease in dogs with access to contaminated water or environmental sources [22, 23]. The patient described in this case had no obvious evidence of systemic disease and lived primarily indoors. Infection source for these reasons was uncertain. Footpad hyperkeratosis and depigmentation in this case could be explained by the chronicity of the disease that may worsen the already existing ones due to inflammation and keratinization abnormalities. Footpad erosion and/or ulceration albeit rarely reported [20, 23] was present in all four limbs and obviously was the cause of the intermittent lameness witnessed.

Therapy applied in this patient with either fluconazole alone or combination of AMB and itraconazole proved effective only for footpad lesions but not for skin nodules. Surgical excision of the nodules was not initially attempted because of their multifocal distribution mainly on the distal extremities. It was estimated that systemic therapy would probably reduce both the size and the number of nodules, thus facilitating their surgical removal later on. However, the systemic therapy administered seems to be ineffective for skin nodules, at least in chronic cases of canine cutaneous protothecosis.

## 4. Concluding Remarks

Although canine protothecosis usually presents with the disseminated form, cutaneous disease as sole clinical manifestation of the infection may also be witnessed.

Systemic therapy seems to be ineffective for skin nodules, at least in chronic cases of canine cutaneous protothecosis.
